# Triethylenetetramine Synergizes with Pharmacologic Ascorbic Acid in Hydrogen Peroxide Mediated Selective Toxicity to Breast Cancer Cell

**DOI:** 10.1155/2017/3481710

**Published:** 2017-02-08

**Authors:** Lianlian Wang, Xiaofang Luo, Cong Li, Yubing Huang, Ping Xu, Laetitia H. Lloyd-Davies, Thibaut Delplancke, Chuan Peng, Rufei Gao, Hongbo Qi, Chao Tong, Philip Baker

**Affiliations:** ^1^Department of Reproduction Health and Infertility, The First Affiliated Hospital of Chongqing Medical University, Chongqing 400016, China; ^2^Department of Obstetrics and Gynecology, The First Affiliated Hospital of Chongqing Medical University, Chongqing 400016, China; ^3^Canada-China-New Zealand Joint Laboratory of Maternal and Fetal Medicine, Chongqing Medical University, Chongqing 400016, China; ^4^Molecular Medicine and Cancer Research Center, Department of Biochemistry and Molecular Biology, Chongqing Medical University, Chongqing 400016, China; ^5^College of Medicine, University of Leicester, Leicester LE1 7RH, UK; ^6^Laboratory of Lipid & Glucose Metabolism, The First Affiliated Hospital of Chongqing Medical University, Chongqing 400016, China; ^7^Laboratory of Reproductive Biology, School of Public Health and Management, Chongqing Medical University, Chongqing 400016, China

## Abstract

Breast cancer is characterized by overexpression of superoxide dismutase (SOD) and downregulation of catalase and more resistance to hydrogen peroxide (H_2_O_2_) than normal cells. Thus, relatively high H_2_O_2_ promotes breast cancer cell growth and proliferation. However, excessive intracellular H_2_O_2_ leads to death of breast cancer cells. In cancer cells, high level ascorbic acid (Asc) is able to be autoxidized and thus provides an electron to oxygen to generate H_2_O_2_. In the present study, we demonstrated that triethylenetetramine (TETA) enhances Asc autoxidation and thus elevates H_2_O_2_ production in MCF-7 cells. Furthermore, Asc/TETA combination significantly impaired cancer cell viability, while having much milder effects on normal cells, indicating Asc/TETA could be a promising therapy for breast cancer. Moreover, SOD1 and N-acetyl-L-cysteine failed to improve MCF-7 cells viability in the presence of Asc/TETA, while catalase significantly inhibited the cytotoxicity of Asc/TETA to breast cancer cells, strongly suggesting that the selective cytotoxicity of Asc/TETA to cancer cells is H_2_O_2_-dependent. In addition, Asc/TETA induces RAS/ERK downregulation in breast cancer cells. Animal studies confirmed that Asc/TETA effectively suppressed tumor growth in vivo. In conclusion, TETA synergizes pharmacologic Asc autoxidation and H_2_O_2_ overproduction in breast cancer cells, which suppresses RAS/ERK pathway and results in apoptosis.

## 1. Introduction

Hydrogen peroxide plays an integral role in cancer cell biology. Cancer cells produce more H_2_O_2_ than normal cells [1], firstly due to an overreaction of enzymes in the electron transport chain that produces excessive reactive oxygen species (ROS) [2] and secondly as a consequence of the overexpression of superoxide dismutase (SOD), which converts superoxide (O_2_^−^) to hydrogen peroxide (H_2_O_2_) [3].

Breast cancer is the leading cause of cancer-related deaths in females worldwide [4]. Like many malignancies it is characterized by overexpression of SOD along with downregulation of catalase (CAT), which converts H_2_O_2_ to H_2_O and O_2_. Thus, breast cancer cells maintain a higher intracellular H_2_O_2_ than normal cells [5], suggesting breast cancer cells are able to accumulate and tolerate H_2_O_2_ within certain range. However, mild elevating of H_2_O_2_ in cancer cells has been shown to arrest the cell cycle and induce apoptosis and has proven beneficial [6, 7]; this indicates selective overload of H_2_O_2_ in cancer cells could be a therapeutic strategy for breast cancer. Indeed, hydrogen peroxide inducible agents have shown potential as anticancer drugs [8]. However, most chemotherapeutic agents for cancer are toxic to the host. Therefore, existing medicine or natural products that selectively promote H_2_O_2_ production in cancer cells, sparing normal cells, are promising candidates for achieving therapeutic activity and selectivity.

Ascorbic acid (Asc), also known as vitamin C, is a well-known natural antioxidant. It has been long assumed to be essential for free radical clearance [9]. Previous studies have reported that high concentrations of Asc are able to induce autoxidation and thus reveal anticancer effects [7], while lower concentrations of Asc failed to show similar effects [10].

In sequential one-electron oxidations, the high concentration of Asc donates 2 electrons to oxygen resulting in formation of dehydroascorbic acid (DHA) and H_2_O_2_. The sequential one-electron oxidation of Asc can occur via the dianion Asc^2−^, which autoxidizes in the presence of dioxide to produce the Asc^−^, dehydroascorbic acid, and H_2_O_2_ [11]. This process is shown in the following formulas:

(1) Ascorbate+H2O⟶AscH−+Asc•−Asc2−+O2⟶Asc•−+O2•−2Asc•−+H+⟶AscH−+DHA2O2•−+2H+⟶H2O2+O2

Therefore, it is important to investigate whether a high concentration of Asc associated autoxidation is critical for its anticancer effects. Asc is very stable and barely autoxidizes alone. However, in the presence of oxidative metal activators, such as iron, copper, and manganese, Asc autoxidation can be dramatically promoted as evidenced by the accumulation of ascorbic acid ion (Asc^•−^) and ultimately result in elevated O_2_^•−^ and H_2_O_2_ production [12]. In addition, O_2_^•−^ can further be reduced to H_2_O_2_ by accepting electron from Asc [13].

**Figure 1:**
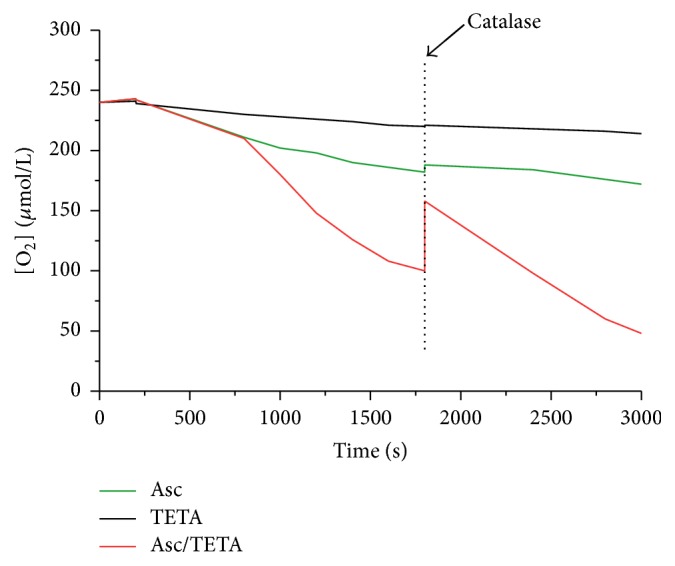
The effects of TETA on Asc oxidation. Oxygen consumption rate (OCR) of Asc in aqueous solution was determined by the use of an electrode oxygen monitor. 1 mM Asc alone, 30 *μ*M TETA/1 mM Asc, or 30 *μ*M TETA alone was added to DMEM with 10% FBS. 600 U/mL catalase was then given into each reaction to determine the return of O_2_.

The function of catalysts for Asc autoxidation in aqueous solution mainly relies on their component groups [7], especially the amino groups, which are considered prooxidative [14]. Triethylenetetramine (TETA) is a telomerase inhibitor that has been clinically used to treat cancer for decades [15]. Previous studies demonstrate that TETA could overcome cisplatin resistance in human ovarian cancer cell cultures, via inhibition of Cu/Zn superoxide dismutase, namely, SOD1 [16, 17]. TETA is an alkali compound containing two amino groups and has a tendency to undergo redox as well as acid-base reactions in aqueous solution. Moreover, it possesses 4 nitrogen atoms, which have 1s22s22p3 electronic arrangement. Each nitrogen atom has a lone pair of electrons, which is able to pair with a proton from Asc. Therefore, we hypothesized that TETA could enhance Asc autoxidation in breast cancer cells and thus elevate intracellular H_2_O_2_, which will further boost Asc derived selective cytotoxicity to breast cancer cells (Figure 1). In the present study, we investigated the effect TETA has on H_2_O_2_ production from Asc solution. We then used in vitro models to study its role in regulating breast cancer cell apoptosis, as well as underlying molecular mechanisms, by the use of an in vivo animal model.

## 2. Materials and Methods

### 2.1. Cell Culture

All the cell lines were purchased from The Cell Bank of Chinese Academy of Sciences (Shanghai). Cells were cultured in Dulbecco's modified Eagle's medium (DMEM, HyClone, SH30022.01B, USA) supplemented with 10% Fetal Bovine Serum (FBS) (Gibco, USA) at 37°C in a humidified atmosphere with 5% CO_2_.

### 2.2. Oxygen Consumption Assay

The rate of oxygen consumption (OCR, *d*[O_2_]/*dt*) was determined as previously described [7]. Briefly, a Clark electrode oxygen monitor (YSI Inc.) was connected to an ESA Biostat multielectrode system in DMEM (10% FBS). The effect of TETA on the OCR of Asc was then measured and recorded. Accumulation of H_2_O_2_ was determined by adding catalase (Sigma, C9322-1G, Germany).

### 2.3. MTT Assay

MCF-7 cells were seeded in 96-well plates (3 × 10^3^ cells/well), followed by 12 h or 24 h of treatments. The media containing Asc was removed before being subjected to MTT assay, because the oxidative products of Asc interfere with the MTT assay. 100 *μ*L of serum-free DMEM medium was applied into each well, and then 20 *μ*L of 3-(4,5)-dimethylthiahiazo(-z-y1)-3,5-diphenytetrazoliumromide (MTT) (Sigma, Germany) was added to each well. Followed by 4 h of incubation at 37°C, all media were removed and then 150 *μ*L of dimethyl sulfoxide (DMSO) (Sigma, Germany) was added to each well; after 10 min of shaking, the value of OD_490_ was recorded by a Varioskan Flash (Thermo Fisher, Finland).

### 2.4. ROS Measurement

The intracellular ROS was detected by using H_2_DCF (Sigma, Germany) as previously reported [18]. Briefly, 10 *μ*M of H_2_DCF was added onto cells for 30 min, and then cells were harvested and analyzed by a fluorescence microscope (KEYENCE Corporation).

### 2.5. Western Blotting

MCF-7 cells were harvested by the use of ice-cold RIPA lysis buffer (Beyotime Biotechnology, Shanghai, China) and protein concentration was determined using the BCA protein quantification kit (Beyotime Biotechnology, Shanghai, China); western blotting was performed as previously described [19]. Primary antibodies of anti-Ac-H3, anti-SOD1, anti-CAT, anti-ERK, anti-p-ERK, anti-Cyt-C, and anti-caspase 9 were purchased from Bioworld Technology (Nanjing, China), anti-PARP, rabbit anti-caspase 3, and anti-Ac-H3 were purchased from Cell Signaling Technology, Danvers, USA, anti-RAS was purchased from BD, USA, and anti-GAPDH was purchased from Earthox, Millbrae, USA. The secondary antibodies were purchased from Biogot Biotechnology (Nanjing, China), and ECL SuperSignal West Femto Maximum Sensitivity Substrate was purchased from Thermo Fisher.

### 2.6. Transient Transfection

1 × 10^5^ cells were plated into each well of a 24-well plate 24 hours before transfection. shRNA (600 ng/well) or Plasmids (800 ng/well) were transfected with Lipofectamine 3000 (Invitrogen, USA) according to the manufacturer's protocol.

SOD1, NM_000454.3-582s1c1: CCGGGCTGTAGAAATGTATCCTGATCTCGAGATCAGGATACATTTCTACAGCTTTTTG.
CAT, NM_001752.2-1371s1c1: CCGGCGGAGATTCAACACTGCCAATCTCGAGATTGGCAGTGTTGAATCTCCGTTTTTG.

### 2.7. Xenograft Study

6-week-old female nude mice (BALB/c-nu) were purchased from Vital River Laboratories (Beijing, China), the animal experiments were approved by the Medical Ethics Committee of Chongqing Medical University, and all of the procedures were in accordance with the National Institutes of Health guide for the care and use of Laboratory animals. In short, MCF-7 cells (5 × 10^6^ in 200 *μ*L) were subcutaneously delivered into the hind leg of mice, and a 0.72 mg 90 days' release 17*β*-estradiol pellet (Innovative Research of America, USA) was implanted subcutaneously into the front-back area to facilitate optimal tumor growth. The tumors were allowed to grow 14 days to reach the greatest dimension of about 3–5 mm, and treatments were initiated on the 14th day. Mice were randomly divided into 4 groups, including control (0.01 M PBS); Asc (3 g/kg body weight); TETA (30 mg/kg body weight); and Asc (3 g/kg body weight) plus TETA (30 mg/kg body weight), 10 mice in each group. Treatments were given via intraperitoneal injection daily for 25 consecutive days. Tumor size was measured every 2 days using a vernier caliper, while tumor volume was estimated based on the following formula: tumor volume (mm^3^) = *L* × *W*^2^/2, where *L* is the greatest dimension of the tumor, and *W* means the dimension of the tumor in the perpendicular direction. Animals were sacrificed by CO_2_ euthanasia when the tumor size reached 1,000 mm^3^.

### 2.8. Statistical Analysis

Data are expressed as mean ± SD. A variety of statistical tests using GraphPad Prism 5 software were used on the basis of the design required for the specific question being asked. This meant using *t*-tests and 2-way ANOVA. A value of *p* < 0.05 was considered statistically significant.

## 3. Results

### 3.1. TETA Synergizes Ascorbic Acid Oxidation

To investigate the effect of TETA on promoting H_2_O_2_ generation from Asc, oxygen consumption of Asc in the presence and absence of TETA has been measured, respectively. As shown in Figure 1, 1 mM Asc in DMEM with 10% FBS resulted in an OCR of 55 nmol/L/s; and additional 30 *μ*M of TETA increased OCR to 110 nmol/L/s, while 30 *μ*M of TETA alone barely consumes O_2_ and generates H_2_O_2_. However, in the presence of catalase (600 U/mL), H_2_O_2_ accumulation in Asc/TETA was dramatically suppressed compared to Asc or TETA alone. Taken together, this evidence strongly suggested that TETA enhanced Asc-dependent H_2_O_2_ generation.

### 3.2. Asc/TETA Combination Promotes Apoptosis in MCF-7 Cells

**Figure 2:**
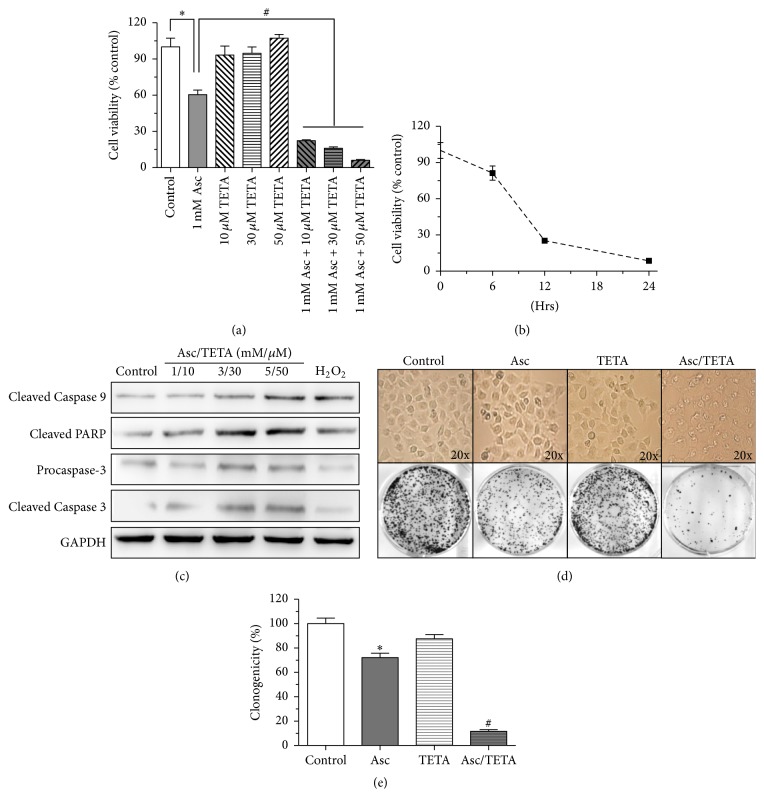
Asc/TETA combination induces apoptosis in MCF-7 cells. MCF-7 cells were treated with 1 mM Asc along with 10 *μ*M, 30 *μ*M, or 50 *μ*M of TETA for 12 hours. (a) MTT assay was performed to assess MCF-7 viability, ^*∗*^*p* < 0.005, ^#^*p* < 0.0001, *n* = 6; (b) viability of MCF-7 cells was measured by MTT assay after 6, 12, and 24 hours of 1 mM Asc/10 *μ*M TETA treatment, *n* = 6. (c) Effects of different dosage of Asc/TETA (1 : 100) on proapoptotic signaling were examined by western blotting; (d) MCF-7 cells cloning formation experiments were performed after 12 hours of 1 mM Asc/10 *μ*M TETA treatment. (e) Statistic analysis of 3 independent experiments, ^*∗*^*p* < 0.05 versus control, ^#^*p* < 0.01 versus Asc, *n* = 3.

To further examine the cytotoxicity of Asc/TETA derived H_2_O_2_ to breast cancer cells, MCF-7 cells were treated with Asc (1 mM) along with different doses of TETA (10 *μ*M, 30 *μ*M, and 50 *μ*M) for 12 hours. MTT assay results demonstrated that Asc alone resulted in a 40% reduction in MCF-7 proliferation, while TETA alone did not show any effect on cell viability. Intriguingly, the combinations of Asc and TETA significantly decreased cell viability compared to Asc alone (Figure 2(a)). Moreover, a prolonged incubation of Asc/TETA for 24 hours led to further reduction in cell viability, indicating that the anticancer effect of Asc/TETA combination is in a time-dose manner (Figure 2(b)). Further western blotting unveiled that Asc/TETA suppresses MCF-7 cell viability by elevating apoptotic signaling of caspase 9 and caspase 3 (Figure 2(c)). Morphology and cell cloning experiments confirmed that TETA synergizes Asc mediated cytotoxicity on MCF-7 cells (Figures 2(d) and 2(e)). Taken together, these results strongly suggest that TETA synergizes the anti-breast cancer effect of Asc in vitro.

### 3.3. Asc and TETA Synergize to Enhance Cytotoxicity In Vitro

**Figure 3:**
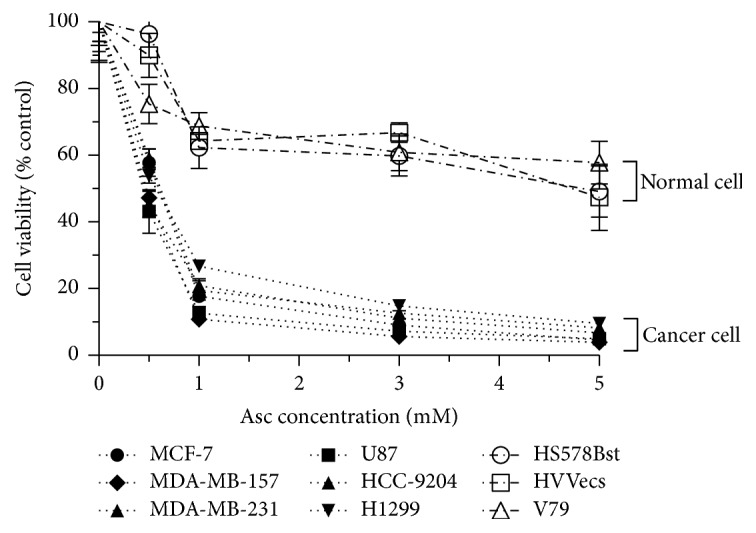
Selective cytotoxicity of Asc/TETA on various cell lines. MCF-7, MDA-MB-157, MDA-MB-231, U87, HCC-9204, H1299, Hs578Bst, HUVEC, or V79 cells were incubated with 1 mM, 3 mM, or 5 mM of Asc, respectively, along with 10 *μ*M, 30 *μ*M, or 50 *μ*M of TETA to maintain 1 : 100 TETA-to-Asc ratio. MTT assay was performed to assess the cytotoxicity of Asc/TETA on different cell lines after 12 hours of incubation; experiment was repeated three times.

To further validate whether the synergistic effects of Asc and TETA on cell death are specific for cancer cells, in addition to various cancer cell lines such as MCF-7, MDA-MB-157, MDA-MB-231, U87, HCC-9204, and H1299, multiple normal cell lines including Hs578Bst, HUVEC, and V79 were incubated with different concentrations of TETA (5 *μ*M, 10 *μ*M, 30 *μ*M, and 50 *μ*M) and corresponding doses of Asc (0.5 mM, 1 mM, 3 mM, and 5 mM) in accordance with the 1 : 100 TETA/Asc ratio. MTT assay results demonstrated that Asc and TETA have synergistic cytotoxicity on cancer cells but much milder effects on the viability of normal cells (Figure 3).

### 3.4. H_2_O_2_ Is Critical for Asc/TETA Induced Cytotoxicity to MCF-7 Cell

**Figure 4:**
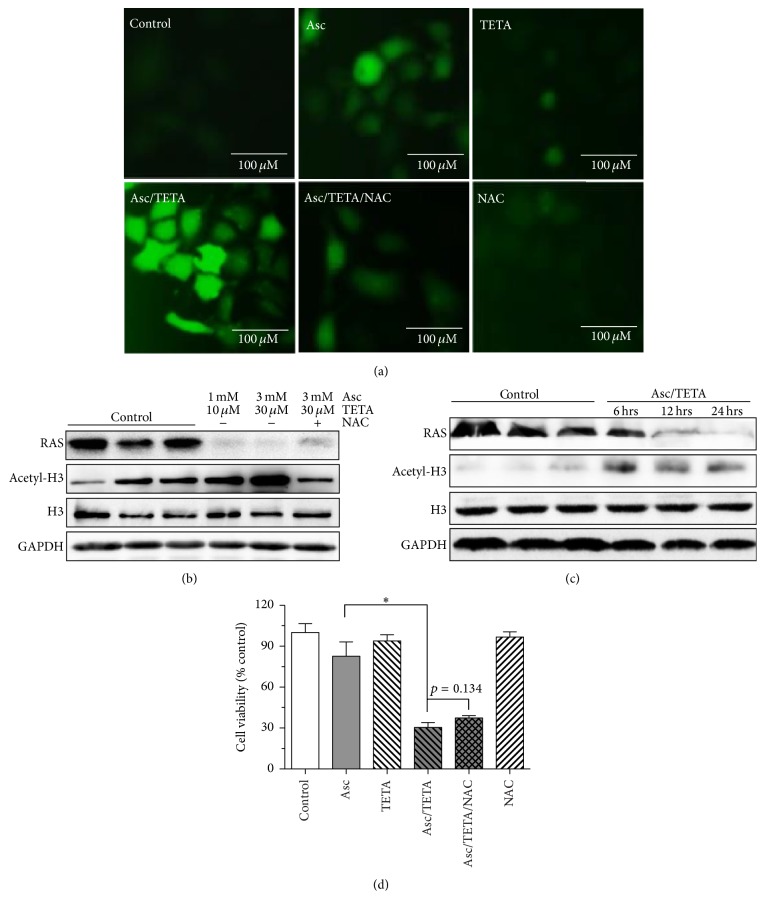
Asc/TETA induces H_2_O_2_-dependent RAS downregulation and apoptosis in MCF-7 cells. MCF-7 cells were treated with 1 mM Asc, 10 *μ*M TETA, 1 mM Asc/10 *μ*M TETA, 5 mM NAC, or 1 mM Asc/10 *μ*M TETA and 5 mM NAC, respectively, for 4 hours. (a) ROS generation in MCF-7 cells was examined by H2DCF staining; (b) MCF-7 cells were treated with 1 mM Asc/10 *μ*M TETA, 3 mM Asc/30 *μ*M TETA, or 3 mM Asc/30 *μ*M TETA with 5 mM NAC for 12 hours. RAS expression and H3 acetylation were assessed by western blotting; (c) MCF-7 cells were treated with 1 mM Asc/10 *μ*M TETA for 6, 12, and 24 hours, respectively; RAS expression and H3 acetylation were assessed by western blotting; (d) MCF-7 cells were treated with 1 mM Asc, 10 *μ*M TETA, 5 mM NAC, 1 mM Asc/10 *μ*M TETA, or 1 mM Asc/10 *μ*M TETA plus NAC for 12 hours; cell viability was measured with MTT assay, ^*∗*^*p* < 0.005, *n* = 4. Experiment was repeated three times.

We then assessed the ROS level in MCF-7 cells. After 4 hours of incubation, Asc alone moderately elevated ROS level in MCF-7 cells, while TETA alone did not show any effect on ROS generation. However, Asc/TETA coincubation resulted in a dramatic increase of ROS compared to the other groups, while in the presence of N-acetyl-L-cysteine (NAC, Sigma, V900429, Germany), which degrade ROS except H_2_O_2_, Asc/TETA showed much less fluorescence staining (Figure 4(a)). Taken together, these data suggested TETA potentiated not only H_2_O_2_ but also other types of ROS production from Asc in MCF-7 cells,

Nevertheless, ROS include hydrogen peroxide (H_2_O_2_), superoxide anions (O_2_^•−^), and hydroxyl radical, to ascertain whether the cytotoxicity of Asc/TETA specifically results from H_2_O_2_; NAC was applied to MCF-7 cells along with Asc/TETA.

Western blotting showed that Asc/TETA combination treatment resulted in inhibition of RAS expression, which were not rescued by extra NAC treatment. Inversely, although Asc/TETA also lead to elevation of H3 acetylation in dose-dependent manner, such effect was totally reversed by 5 mM NAC (Figure 4(b)). Moreover, the effects of Asc/TETA on RAS expression and H3 acetylation demonstrated a time-dependent manner (Figure 4(c)). The data suggests that TETA/Asc suppresses RAS expression in MCF-7 cells principally through H_2_O_2_, while it upregulates H3 acetylation mainly through the other types of ROS. Most importantly, 5 mM NAC failed to eliminate the cytotoxicity caused by Asc/TETA. Taken together, these results indicate that H_2_O_2_ is the primary cytotoxic ROS induced by Asc/TETA in MCF-7 cells and possibly through the inhibition of RAS expression.

### 3.5. The Selective Cytotoxicity of Asc/TETA Derived H_2_O_2_ to MCF-7 Cells due to Compromised CAT Expression

**Figure 5:**
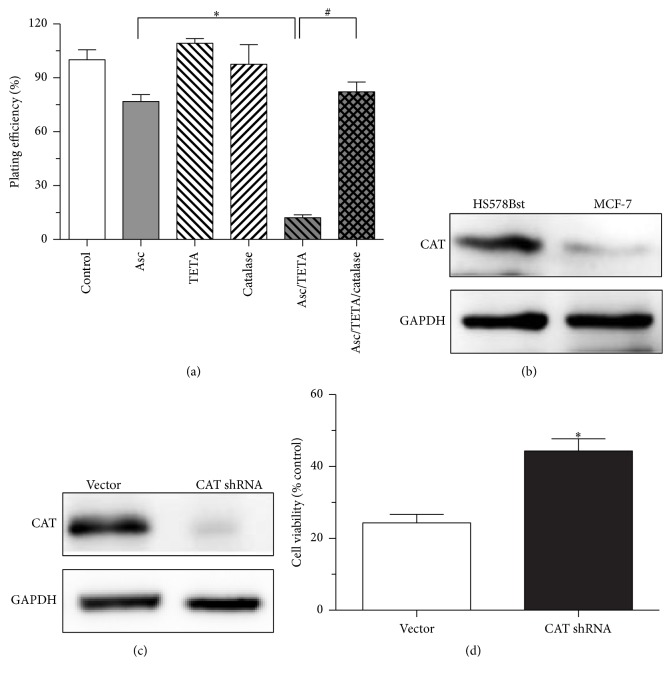
CAT expression levels in breast cancer cells and normal cells. (a) MCF-7 cells were treated with 1 mM Asc, 10 *μ*M TETA, 300 U/mL catalase, 1 mM Asc/10 *μ*M TETA, or 1 mM Asc/10 *μ*M TETA plus catalase for 12 hours; cloning formation assay was performed, ^*∗*^*p* < 0.0001, ^#^*p* < 0.0001, *n* = 4; (b) CAT expression levels in HS578Bst and MCF-7 cells were determined by western blotting; (c) CAT expression in HS578Bst was suppressed by CAT shRNA; (d) HS578Bst cell viability after CAT shRNA treatment was measured by MTT assay, *p* < 0.005, *n* = 4.

We subsequently investigated the mechanism underlying the discrepancy of the cytotoxicity of Asc/TETA combination in cancer cells and normal cells. The effect of catalase on Asc/TETA induced cell death was examined by clone formation experiment; the data (Figure 5(a)) shows that Asc alone moderately reduced plating efficiency of MCF-7 cells, while neither TETA nor catalase shows similar effect. However, Asc/TETA combination significantly suppressed plating efficiency of MCF-7 cells, but the reduction was almost fully restored in the presence of catalase. We then determined the expression levels of CAT in MCF-7 cells and normal cells by immunoblotting. It was shown that CAT expression level is significantly compromised in MCF-7 cells compared to the normal cells (Figure 5(b)). To further determine the importance of CAT in resistance to Asc/TETA induced cell death, shCAT was transfected into the normal breast epithelial HS578Bst cells; it only dramatically repressed CAT expression in HS578Bst cells (Figure 5(c)) but also significantly impaired cell viability (Figure 5(d)). This strongly suggests that the selective toxicity to cancer cells could be attributed to CAT downregulation, and thus the cytotoxicity of combined TETA and Asc use is primarily mediated by H_2_O_2_.

### 3.6. Asc/TETA Induced Cytotoxicity to MCF-7 Cells Is Not Mediated by O_2_^•−^

**Figure 6:**
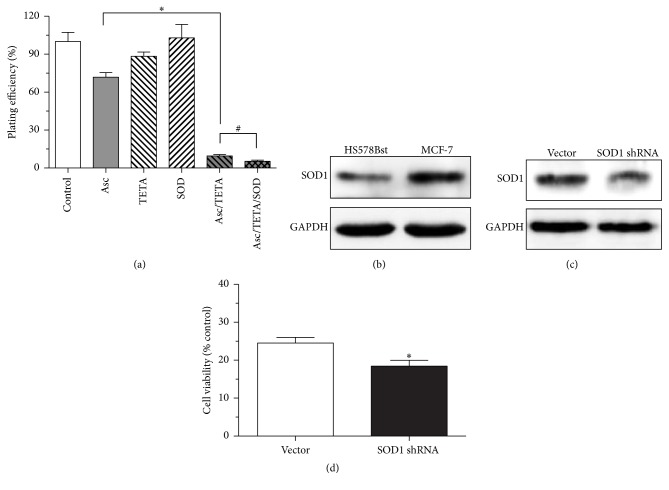
The effects of SOD on Asc/TETA induces apoptosis. (a) 1 mM Asc, 10 *μ*M TETA, 100 U/mL SOD1, 1 mM Asc/10 *μ*M TETA, or 1 mM Asc/10 *μ*M TETA plus SOD was applied onto MCF-7 cells for 12 hours. Cloning formation assay was performed to determine the plating efficiency. ^*∗*^*p* < 0.0001, ^#^*p* < 0.05, *n* = 4; (b) SOD1 expression levels in HS578Bst and MCF-7 cells were assessed by western blotting; (c) downregulation of SOD1 expression in MCF-7 cells by SOD1 shRNA was determined by western blotting; (d) MCF-7 cell viability after SOD1 shRNA treatment was measured by MTT assay, ^*∗*^*p* < 0.05, *n* = 4.

Although H_2_O_2_ has been found critical for TETA/Asc mediated cytotoxicity, the possibility of O_2_^•−^ being involved in such anticancer effects has yet to be ruled out. To distinguish the roles of O_2_^•−^ and H_2_O_2_ in TETA/Asc combination derived cytotoxicity, 100 U/mL SOD was applied onto MCF-7 cells with 1 mMAsc and 10 *μ*M TETA. In contrast to CAT, SOD failed to rescue cell survival in the presence of Asc and TETA but exacerbated Asc and TETA combination induced cell death (Figure 6(a)). This indicates that O_2_^•−^ does not contribute to TETA and Asc mediated cytotoxicity to cancer cells. Not surprisingly, SOD protein expression in MCF-7 cells was significantly higher than normal cells (Figure 6(b)). To further confirm this finding, SOD1 in MCF-7 cells was knocked down by shRNA (Figure 6(c)). In combined Asc/TETA treatment, shSOD1 did not result in enhanced viability of MCF-7 cell but led to more cell death (Figure 6(d)). This data suggests that the selective cytotoxicity of Asc/TETA to MCF-7 cells is not mediated by O_2_^•−^.

### 3.7. The Signaling Associated with Asc/TETA Treatment in Cancer Cells

**Figure 7:**
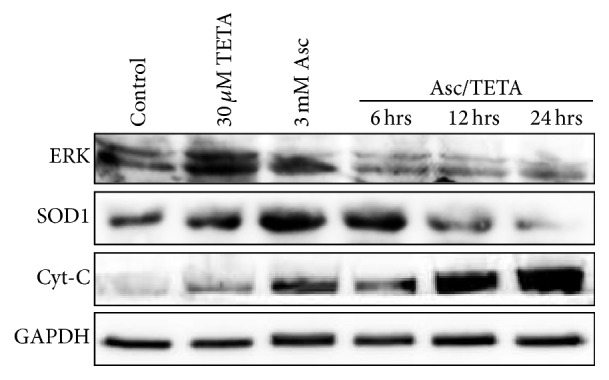
The effects of Asc/TETA on ERK and SOD expression. MCF-7 cells were incubated with 3 mM Asc, 30 *μ*M TETA, or 3 mM Asc/30 *μ*M TETA for various duration; ERK, RAS, and Cyt-C protein levels were assessed by western blotting.

We then investigated which signaling pathways are involved in Asc/TETA induced cell death. As shown in Figure 7, western blots illustrated that Asc/TETA treatment suppressed ERK1/2 and SOD1 expression in MCF-7 cells. To further determine the type of cell death caused by Asc and TETA, Cytochrome C (Cyt-C), the key regulator of apoptosis, has been measured. Asc/TETA induced Cyt-C release was time dependent, which is consistent with previous MTT results. These data strongly suggest that Asc/TETA induced cancer cell apoptosis is probably mediated by RAS-ERK pathway.

### 3.8. TETA Enhances Asc-Induced Cytotoxicity In Vivo

**Figure 8:**
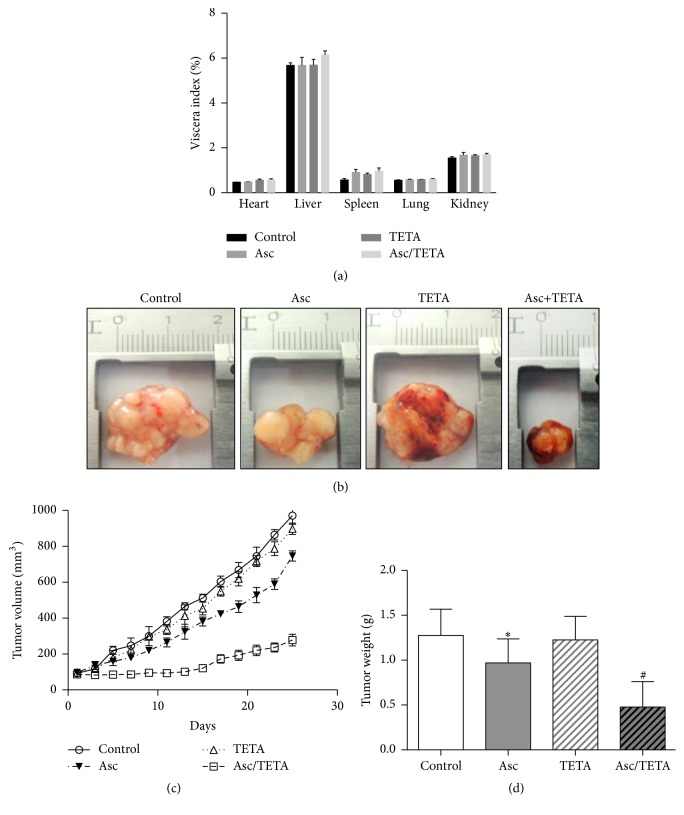
Asc/TETA suppresses tumorigenesis in mouse breast cancer model. 5 × 10^6^ MCF-7 cells were subcutaneously delivered into the hind leg of female nude mice. 14 days later, mice were randomly divided into 4 groups and given vehicle (0.01 M PBS); Asc (3 g/kg body weight); TETA (30 mg/kg body weight); Asc (3 g/kg body weight) plus TETA (30 mg/kg body weight), respectively, via intraperitoneal injection once a day. On the 25th day of treatment, (a) viscera index; (b) representative pictures of tumor from different groups; (c) tumor volume, *p* < 0.0001, *n* = 6; (d) tumor weight ^*∗*^*p* < 0.05, ^#^*p* < 0.005, *n* = 6.

In order to examine whether combined Asc/TETA has cytotoxicity to tumor cells in vivo, the combination was administrated into nude mice with transplanted tumors. After 25 days of treatment, there was no significant difference in viscera index between the 4 groups as has been observed (Figure 8(a)), indicating Asc/TETA has low toxicity for these organs. However, there was difference in the day 25 tumor volume between the groups, 971.1 ± 24.20 mm^3^ in control group, 898.0 ± 16.03 mm^3^ in TETA group, and 746.5 ± 14.44 mm^3^ in Asc group, but it significantly reduced to 278.0 ± 16.42 mm^3^ in Asc/TETA group. In congruence, the tumor weights were 1.276 ± 0.097 g, 0.969 ± 0.095 g, 1.226 ± 0.087 g in control, Asc, and TETA group, respectively, but dramatically declined to 0.478 ± 0.094 g by Asc/TETA treatment (Figures 8(b), 8(c), and 8(d)). These results indicate that TETA and Asc have synergistic antitumor effects in vivo, without notable toxicity to internal organs.

## 4. Discussion

Given that one of the typical characteristics of breast cancer is SOD overexpression, along with compromised CAT expression, the intracellular oxidative stress is higher in the cancer cells compared to normal cells [20]. Wlassoff et al. have shown that hydroxyl radicals derived from H_2_O_2_ promote breast cancer cell apoptosis in the presence of a tamoxifen–ferrocene conjugate [21]. Other groups have demonstrated that the excessive H_2_O_2_ production and accumulation in cancer cells could trigger cancer cell cycle arrest and apoptosis [6], suggesting the enhancement of intracellular H_2_O_2_ concentration could be a promising therapy for breast cancer. Therefore, a natural compound which is able to selectively induce H_2_O_2_ generation in cancer cells could be an ideal therapy for breast cancer.

Asc is a potent natural antioxidant that has long been assumed to be beneficial for cancer treatment. Intriguingly, it has been reported that, in cancer cells, Asc is oxidized by donating an electron, which had an oxidation reaction with the metal ion (copper, iron, and manganese), producing the anticancer H_2_O_2_ [12]. Another group has also shown that 4 h of 1 mM Asc treatment on C6 cells triggers intracellular Cu release [22], which can further promote Asc oxidation, indicating H_2_O_2_ generated by Asc autoxidation might be beneficial for cancer treatment. However, the effectiveness of Asc derived H_2_O_2_ on treating cancer remains debatable. Firstly, there is no strong association between plasma Asc concentration and breast cancer risk [23]; secondly, H_2_O_2_ has been found to enhance growth of breast cancer [5]. Both of them indicate that not only is a high dose of Asc needed to induce a dramatic elevation of intracellular H_2_O_2_ flux, but also a reagent that could potentiate or promote H_2_O_2_ production from Asc autoxidation might be critical for Asc clinically utilized in breast cancer therapy.

Considering human circulating Asc concentration is relative high, ranging from 20 to 80 *μ*mol/L [24, 25], and quite stable in various countries [26], it is reasonable to pursue the effects of pharmacologic Asc on breast cancer. Indeed, cumulative evidence indicates that high doses of Asc could be beneficial for breast cancer treatment. Yun et al. recently reported that high dose Asc selectively kills KRAS and BRAF mutant colorectal cancer cells [27]. Similarly, another group has also shown that pharmacologic concentrations of Asc inhibit proliferation and induce apoptosis in various colorectal cancer lines, probably by promoting intracellular oxidative stress [28]. Asc has also exhibited anticancer properties in pancreatic cancer cells [29], providing further evidence to suggest that high dose of Asc induces H_2_O_2_ flux in the presence of catalytic metal ions, resulting in oxidative stress in cancer cells which ultimately leads to apoptosis. These results suggest that pharmacologic Asc may be needed to cause breast cancer cell death. Our data confirmed that a high dose of Asc is essential for inducing MCF-7 cell apoptosis in vitro and inhibiting tumorigenesis in vivo. Although many studies have shown Asc can be used as an adjuvant for breast cancer chemotherapy [30], therapy that primarily depends on Asc-induced oxidative stress in cancer cells has yet to be investigated.

TETA is a charge-deficient isosteric analogue of spermidine and a Cu (II) chelating compound. It is usually used for treating Wilson's disease. Recently, its potential in treating cancer has been unveiled; growing evidence demonstrates that TETA plays a role against cancer by inhibiting telomerase [31], in antiangiogenesis [32], and in suppressing cancer cell proliferation by modulating metabolism [33], as well as in induction of cell withered death [34]. However, none of these studies focused on the effect of TETA's chemical properties on cancer, specifically the two amino groups, and their role as a potential catalyst for Asc autoxidation. Our work is the first to report that, in aqueous solution, TETA enhances H_2_O_2_ production by increasing Asc oxidation. Thereafter, we examined whether Asc/TETA combination could sufficiently lead to breast cancer cell death. In vitro and in vivo experiments consistently demonstrated that high dose Asc alone only resulted in mild toxicity to cancer cells, while TETA alone barely shows any effects. However, Asc/TETA combination significantly inhibited MCF-7 cell viability, strongly suggesting that Asc/TETA combination could be a promising neoadjuvant therapy for breast cancer. Nevertheless, the underlying molecular mechanism remains to be discovered.

To examine the cytotoxicity of Asc/TETA to breast cancer cells, fluorescent staining of ROS has been performed by the use of H2DCF. The results suggested that Asc/TETA significantly promoted ROS generation in cancer cells. We then determined which type of ROS is responsible for Asc/TETA induced cancer cell death. Adding NAC does not inhibit the cytotoxicity of Asc/TETA, strongly indicating that H_2_O_2_ is the major type of ROS derived from Asc/TETA and the cause of the selective cytotoxicity to breast cancer cell. However, many studies have shown that chronic high extracellular H_2_O_2_, over several months, promotes breast cancer cell proliferation and results in an aggressive phenotype [5, 35, 36]. Nevertheless, our results are consistent with Wlassoff et al., who reported that H_2_O_2_ is the hydroxyl radical which stimulates apoptosis in tamoxifen–ferrocene conjugate treated MCF-7 cells [21]. This indicates that the best therapeutic window of H_2_O_2_-dependent breast cancer treatment would be no longer than 6 months. However, in future studies it would be pertinent to perform a real time measurement of intracellular H_2_O_2_ flux under Asc/TETA treatment as previously reported [37].

It is quite interesting to understand whether TETA/Asc is selectively toxic to cancer cells. It has previously been demonstrated that Asc arrested growth of some cancer cell lines, like HeLa, SK-BR-3, SK-BR-3-Dox, L929, and Mel B16, but did not influence the growth of others: Hef, OVCAR, HEp2, HEp2VA3, and V79 [38]. Our results have shown that Asc/TETA is selectively toxic to cancer cell lines, including MCF-7, MDA-MB-157, MDA-MB-231, U87, HCC-9204, and H1299, but has much milder toxicity to normal cell lines such as Hs578Bst, HUVEC, and V79. Taken together, these data suggest Asc/TETA selectively kill certain type of cancer cells, including the ER-positive MCF-7 breast cancer cells.

To investigate the mechanism of the selective cytotoxicity of Asc/TETA in breast cancer cell, the expression of SOD1 and CAT was modulated using shRNA. Interestingly, downregulation of SOD1 failed to ameliorate the cytotoxicity of Asc/TETA to cancer cells; in contrast, overexpression of catalase effectively halted the cancer cell death induced by Asc/TETA. This confirmed that H_2_O_2_ rather than O_2_^•−^ is the causative factor for Asc/TETA induced cell death. In addition, to further study whether the disturbed expression pattern of SOD1 and CAT in breast cancer cell is related with cell viability, SOD1 and CAT were knocked down by shRNA, respectively. SOD1 is overexpressed in the MCF-7 cell compared to normal cells, and shSOD1RNA treatment resulted in significant reduction in viability of the MCF-7 cell, indicating endogenous SOD1 is critical for maintaining breast cancer cell physiological function. Probably a product of the SOD1, H_2_O_2_, is an important signaling molecule for cancer cells. It is known that certain levels of H_2_O_2_ promote cancer cell proliferation and growth [5]. Furthermore, due to the low levels of expression of CAT in MCF-7 cells, we then downregulated CAT in HS578Bst cells, which demonstrated augmented cell viability. This suggests low level of CAT may be beneficial for cells, possibly by blocking the removal of H_2_O_2_ and thus maintaining a beneficial intracellular H_2_O_2_ concentration.

Numerous studies have shown that RAS-ERK pathway is involved in breast cancer cell growth and metastasis [39, 40]. Our data also suggested that Asc/TETA suppressed RAS and ERK expression in a time- and dose-dependent manner. It also suppressed subsequent cancer cell apoptosis, as evidenced by release of Cyt-C and caspases cleavage, while neither TETA nor Asc alone showed similar effects. In this work, we also observed Asc/TETA treatment result in global H3 acetylation in MCF-7 cells, which is not associated with H_2_O_2_ production and the viability of MCF-7 cells, but evidence has shown that elevating histone acetylation by SIRT1 inhibition induces breast cancer cell apoptosis [41], possibly through the upregulation of p21 [42]. Therefore, it will be interesting to further elucidate whether histone modification in the promoter region of specific genes is involved in Asc/TETA induced breast cancer cell apoptosis. Moreover, we found that Asc/TETA treatment also leads to a downregulation of SOD1 in MCF-7 cells, which is in accordance with the SOD1 shRNA experiment result, indicating the anti-breast cancer effects of Asc/TETA combination are partially mediated by the inhibition of SOD1. Despite other studies also suggesting Asc may be involved in regulating breast cancer cell proliferation and migration through Akt and RhoA, respectively [43, 44], it is unclear whether Asc/TETA is effective through the same pathways and warrants further investigation.

Xenograft experiments validated our findings from the cell model. Administration of Asc/TETA dramatically reduced tumor size and weight, while visceral organs in the nude mice remained unaffected. This result confirmed that TETA synergizes Asc's anti-breast cancer effects in vivo. However, the dose used in this experiment is 3 grams per kg body weight, which is a far higher dose of Asc compared to what has been used previously. Further pharmacodynamics and pharmacokinetics studies for Asc/TETA are needed to explore the optimal administrative approach and dosage for breast cancer treatment. Likewise, a treatment of Asc in combination with another natural mixture, including lysine, proline, and green tea extract, significantly reduced tumor weight and inhibited metastasis to lung, spleen, liver, kidney, and heart in a breast cancer murine model [45]. Similarly, a natural antioxidant mixture containing Asc has also been shown to effectively repress radiation-induced carcinogenesis in animal experiments [46]. It is also reported that oral administration of high dose of Asc represents strong anticarcinogenic effect and prolongs survival time of a rat cancer model induced by benzo[a]pyrene [47].

In addition to the H_2_O_2_ mediated apoptotic effects of Asc/TETA on cancer cells, the synergistic antioxidant properties hold potential in preventing normal cell damage from high intercellular/intracellular ROS. Findings from other groups suggest these additional antitumorigenic effects may be mediated by inhibiting HIF1 [48]. However, evidence suggests that pharmacological Asc might have side effects. Asc induced procoagulant and prothrombotic activation of RBCs, and increased thrombosis has been reported in vivo. RBCs from cancer patients exhibited increased sensitivity to the prothrombotic effects of Asc, reflecting that intravenous gram-dose vitamin C therapy needs to be carefully revisited [49]. An Asc derivative, 6-deoxy-6-chloro-ascorbic acid, exhibited more potent effects in suppressing cancer cell proliferation and is more likely to be used clinically to treat breast cancer [38].

In conclusion, the present work demonstrates that TETA synergizes with pharmacologic Asc in breast cancer to enhance hydrogen peroxide production, thus inhibiting tumorigenic RAS/ERK signaling pathways and inducing apoptosis.
